# Plant Antimicrobial Peptides as Potential Anticancer Agents

**DOI:** 10.1155/2015/735087

**Published:** 2015-03-01

**Authors:** Jaquelina Julia Guzmán-Rodríguez, Alejandra Ochoa-Zarzosa, Rodolfo López-Gómez, Joel E. López-Meza

**Affiliations:** ^1^Centro Multidisciplinario de Estudios en Biotecnología, Facultad de Medicina Veterinaria y Zootecnia, Universidad Michoacana de San Nicolás de Hidalgo, Km 9.5 Carretera Morelia-Zinapécuaro, Posta Veterinaria, 58893 Morelia, MICH, Mexico; ^2^Instituto de Investigaciones Químico Biológicas, Universidad Michoacana de San Nicolás de Hidalgo, Edif. B1, Ciudad Universitaria, 58030 Morelia, MICH, Mexico

## Abstract

Antimicrobial peptides (AMPs) are part of the innate immune defense mechanism of many organisms and are promising candidates to treat infections caused by pathogenic bacteria to animals and humans. AMPs also display anticancer activities because of their ability to inactivate a wide range of cancer cells. Cancer remains a cause of high morbidity and mortality worldwide. Therefore, the development of methods for its control is desirable. Attractive alternatives include plant AMP thionins, defensins, and cyclotides, which have anticancer activities. Here, we provide an overview of plant AMPs anticancer activities, with an emphasis on their mode of action, their selectivity, and their efficacy.

## 1. Introduction

Cancer is a leading cause of death worldwide. In 2012, cancer caused 8.2 million deaths, and cancers of the lungs, liver, colon, stomach, and breast are main types [1]. A hallmark of cancer is the rapid growth of abnormal cells that extend beyond their usual limits and invade adjoining parts of the body or spread to other organs, a process known as metastasis. Cancer treatment requires careful selection of one or more therapeutic modalities, such as surgery, radiotherapy, or chemotherapy. Despite progress in anticancer therapies, the chemotherapeutic drugs used in cancer treatment have the serious drawback of nonspecific toxicity. Additionally, many neoplasms eventually become resistant to conventional chemotherapy because of selection for multidrug-resistant variants [2]. These limitations have led to the search for new anticancer therapies. An attractive alternative is the use of antimicrobial peptides or AMPs, which represent a novel family of anticancer agents that avoid the shortcomings of conventional chemotherapy [3].

AMPs are amphipathic molecules produced by a wide variety of organisms as part of their first line of defense (eukaryotes) or as a competition strategy for nutrients and space (prokaryotes) [4]. Currently, over 2400 AMPs are reported in The Antimicrobial Peptide Database (URL http://aps.unmc.edu/AP/main.php) [5]. The continuous discovery of new AMP groups in diverse organisms has made these natural antibiotics the basic elements of a new generation of potential biomedical treatments against infectious diseases in humans and animals. Moreover, the broad spectrum of biological activities and the low incidence of resistance to these molecules suggest a potential benefit in cancer treatment, which reinforces the importance of their study [6].

AMPs are usually short peptides (12–100 aa residues), which mainly have a positive charge (+2 to +9), although there are also neutral and negatively charged molecules [7]. AMPs are classified into the following four groups according to their structural characteristics: (1) cysteine-rich and *β*-sheet AMPs (*α*- and *β*-defensins); (2) AMPs possessing *α*-helices (LL-37 cathelicidin, cecropins, and magainins); (3) AMPs with extended structure (rich in glycine, proline, tryptophan, arginine, and/or histidine); (4) peptide “loop,” which have a single disulfide bond (bactenecin) [8]. In recent years, several reviews on the structures, mechanisms of action, and emergence of resistance to AMPs have been published, to which the reader is referred for additional information [9–11]. Furthermore, recent reviews of the anticancer activities and selectivity and efficacy of AMPs, particularly from animals, have been reported [12–15]. The mechanisms by which AMPs kill cancerous cells are poorly understood although evidences indicate that both membranolytic and nonmembranolytic mechanisms are involved. The membranolytic activity of AMPs depends on their own characteristics as well as of the target membrane [13]. Also, the selectivity of some AMPs against cancer cells has been related with the charge of membrane, which has a net negative charge [12]. Anionic molecules (phosphatidylserine, O-glycosylated mucins, sialylated gangliosides, and heparin sulfate) confer a net negative charge to cancer cells, which contrasts with the normal mammalian cell membrane (typically zwitterionic) [14, 15]. On the other hand, the nonmembranolytic activities of AMPs involve the inhibition of processes such as angiogenesis, which is essential for the formation of tumor-associated vasculature [14].

Despite the promising characteristics of anticancer agents such as AMPs, only a few of them have been tested using* in vivo* models. Cecropin B from* Hyalophora cecropia* increases the survival time of mice bearing ascitic murine colon adenocarcinoma cells [16]. In the same way, when magainin 2 was tested against murine sarcoma tumors, animals increase its life span (45%) [17]. However, there is little information related to the anticancer effects of plant AMPs. Here, we provide an overview of plant AMP anticancer activities with an emphasis on their mode of action, selectivity, and efficacy. We focus on the anticancer activity reported only for the defensins, thionins, and cyclotides because the cytotoxic effects of these families have been widely described.

## 2. Plant AMPs

Plants are a major source of diverse molecules with pharmacological potential. Over 300 AMP sequences have been described [5]. Plants produce small cysteine-rich AMPs as a mechanism of natural defense, which may be expressed constitutively or induced in response to a pathogen attack. Plant AMPs are abundantly expressed in the majority species, and small cysteine-rich AMPs may represent up to 3% of the repertoire of plant genes [18]. Plant AMPs are produced in all organs and are more abundant in the outer layer, which is consistent with their role as a constitutive host defense against microbial invaders attacking from the outside [19, 20]. Plant AMPs are released immediately after the infection is initiated. AMPs are expressed by a single gene and therefore require less biomass and energy consumption [19, 20]. The majorities of plant AMPs have a molecular weight between 2 and 10 kDa, are basic, and contain 4, 6, 8, or 12 cysteines that form disulfide bonds conferring structural and thermodynamic stability [21]. Plant AMPs are classified based on the identity of their amino acid sequence and the number and position of cysteines forming disulfide bonds. Twelve families have been described, which are listed in Table 1 [21–23].

The primary biological activities of plant AMPs are antifungal, antibacterial, and against oomycetes and herbivorous insects [32, 34, 35]. Additionally, plant AMPs also exhibit enzyme inhibitory activities [36] and have roles in heavy metal tolerance [37], abiotic stress [38], and development [39]. In addition, some plant AMPs show cytotoxic activity against mammalian cells and/or anticancer activity against cancer cells from different origins [25, 28, 31, 40–56]. Of the 12 plant AMP families, 3 contain members with cytotoxic and anticancer properties, the defensins, thionins, and cyclotides. Here, the cytotoxic properties of these peptides are described and the possibility of their use in cancer treatment is discussed.

## 3. Thionins

Thionins were the first AMP isolated from plants [57]. These AMPs belong to a rapidly growing family of biologically active peptides in the plant kingdom and are small cysteine-rich peptides (~5 kDa) with toxic and antimicrobial properties [58]. Thionins are divided into at least four different types depending on the net charge, the number of amino acids, and the disulfide bonds present in the mature protein [59]. Type 1 thionins are highly basic and consist of 45 amino acids, eight of which are cysteines, forming four disulfide bonds. Type 2 thionins consist of 46 or 47 amino acid peptides, are slightly less basic than type 1 thionins, and also have four disulfide bonds. Type 3 thionins consist of 45 or 46 amino acid peptides with three or four disulfide bonds and are as basic as type 2 thionins. Finally, type 4 thionins consist of 46 amino acid peptides with three disulfide bonds and are neutral [58].

The primary role for thionins is plant protection against pathogens [57, 59]. However, they also participate in seed maturation, dormancy, or germination [58], as well as the packaging of storage proteins into protein bodies, or in their mobilization during germination [60]. In addition, thionins may play a role in altering the cell wall upon penetration of the epidermis by fungal hyphae or act as a secondary messenger in signal transduction [61].

### 3.1. Cytotoxic and Anticancer Activity of Thionins

In addition to the activities described, several plant thionins show cytotoxic and anticancer activities (Table 2). The pyrularia thionin from mistletoe (*Pyrularia pubera*) showed an anticancer activity against cervical cancer cells (HeLa) and mouse melanoma cells (B16) with an IC_50_ of 50 *μ*g/mL (half maximal inhibitory concentration); however, the pyrularia thionin is cytotoxic because it causes hemolysis [24]. The anticancer effect is attributable to a cellular response that involves the stimulation of Ca^2+^ influx coupled to depolarization of the plasma membrane, which leads to the activation of an endogenous phospholipase A_2_ and, as consequence, membrane alteration, and finally the cell death.

**Figure 1:**
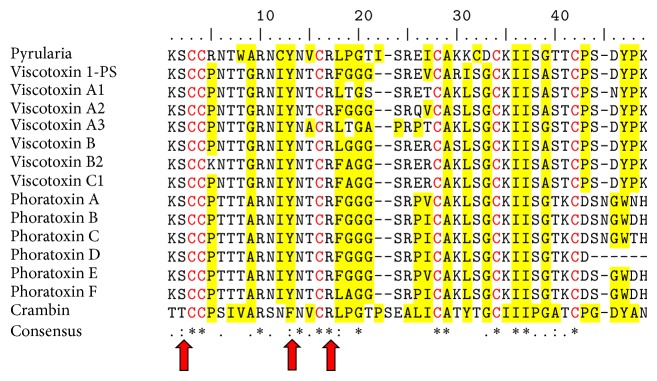
Alignment of amino acid sequences from thionins. The asterisk indicates amino acids conserved in all family members. The cysteine residues present in all sequences and relevant to the classification are indicated in red letters. The red arrows indicate the three residues that are essential for binding to the head regions of the membrane lipids. The hydrophobic residues are shaded in yellow. The thionin sequences included in the alignment were pyrularia (GenBank accession P07504) from* Pyrularia pubera*, viscotoxins 1-PS (GenBank accession P01537), A1 (GenBank accession 3C8P_A), A2 (GenBank accession P32880), A3 (GenBank accession VTVAA3), B (GenBank accession 1JMP_A), B2 (GenBank accession 2V9B_B), and C1 (GenBank accession P83554) from* Viscum album*, phoratoxins A (GenBank accession P01539), B, C, D, E, and F [24] from* Phoradendron tomentosum,* and crambin (GenBank accession P01542) from* Crambe hispanica.*

Another group of thionins with anticancer and cytotoxic activity are the viscotoxins from* Viscum* spp. Viscotoxin B2 showed anticancer activity against rat osteoblast-like sarcoma (IC_50_ 1.6 mg/L) [42]. On the other hand, viscotoxins A1, A2, A3, and 1-PS were cytotoxic to human lymphocytes, due the fact that they induce the production of reactive oxygen species (ROS) and cell membrane permeabilization [26]. Furthermore, a mixture of viscotoxins (50 *μ*g/mL) induced apoptosis in human lymphocytes by activating caspase 3 [43]. Conversely, viscotoxins are far less hemolytic than other thionins. Under the same experimental conditions, pyrularia thionin (20 *μ*g/mL) lysed 50% of human erythrocytes, whereas viscotoxin B (100 *μ*g/mL) lysed only 10% [62]. An alignment of the amino acids sequences of both thionins shows that pyrularia has more hydrophobic amino acids compared to the viscotoxin B (Figure 1). These differences could explain the differential hemolytic activity of both thionins because greater hydrophobicity increases the hemolytic activity of AMPs [63].

Another thionin with anticancer activity is the ligatoxin B (*Phoradendron league*). This AMP (100 *μ*g/mL) inhibited the growth of lymphoma cells (U937GTB) and human adenocarcinoma (ACHN). Ligatoxin B has a DNA binding domain, which may be related to the inhibition of nucleic acid and protein synthesis [28]. Unfortunately, the cytotoxic effects of ligatoxin B have not yet been tested on normal cells.

Several thionins (phoratoxins A–F) have been identified in* Phoradendron tomentosum*, all of which possess toxic activity. Phoratoxins A and B are toxic to rats at doses of 0.5–1 mg/kg, and their mechanism of action is related to changes in the electrical charge and the mechanical activity of the rat papillary muscle [30]. Furthermore, phoratoxins C–F showed differential anticancer activity against different types of solid tumor cells (NCI-H69, ACHN, and breast carcinoma) and hematological tumors (RPMI 8226-S and U-937 GTB). Phoratoxin C was the most toxic with an IC_50_ of 0.16 *μ*M, whereas phoratoxin F had an IC_50_ value of 0.40 *μ*M. Furthermore, phoratoxin C was tested on primary cultures of tumor cells from patients and showed selective activity to breast cancer cells from solid tumor samples. These cells were 18 times more sensitive to phoratoxin C than the hematological tumor cells [31]. These data suggest that these compounds are an alternative for developing a new class of anticancer agents with improved activity against solid tumor malignancies. Despite the marked differences in the activity of phoratoxins, they have a high percentage of identity (~90%) (Figure 1). The small changes in specific amino acids could be the key to the biological activity of these thionins; however, further studies are necessary.

Another thionin with anticancer activity against cancer cell lines is the Thi2.1 thionin from* Arabidopsis thaliana*, which was expressed in a heterologous system [32]. The conditioned media from cells that express Thi2.1 inhibited the viability of MCF-7 cells (94%), A549 (29%), and HeLa cells (38%); however, Thi2.1 also showed cytotoxicity against bovine mammary epithelial cells (89%) and bovine endothelium (93%). The mechanism of action of Thi2.1 has not yet been determined.

In summary, the cytotoxic activity of thionins is not selective; however, these peptides can be exploited for the design of new anticancer molecules. Further investigations are necessary to determine the clinical potential of this class of compounds.

## 4. Plant Defensins

Plant defensins are a class of plant AMPs with structural and functional properties that resemble the defense peptides produced by fungi, invertebrates, and vertebrates, called “defensins.” This group of AMPs has great diversity in amino acid sequence, but its members show a clear conservation of some amino acid positions. This variation in the primary sequence is associated with the diversity of biological activities of plant defensins, which include antifungal and antibacterial activities, in addition to proteinase or amylase inhibitory activities [20]. Plant defensins can form three to four disulfide bridges that stabilize their structure [64]. Studies of the three-dimensional structure of plant defensins have shown that these peptides consist of an *α*-helix and three antiparallel *β*-sheets, arranged in the configuration *βαββ* [19]. These AMPs are classified into two types depending on the structure of the precursor protein from which they are derived. Type 1 defensins are the largest group, and the majority of members contain a signal peptide in the prepeptide sequence linked to the mature defensin at the N-terminus. Type 2 defensins include plant defensins for which the precursor has a signal peptide, the active domain of the defensin, and a C-terminal prodomain [20]. Recently, it was demonstrated that the C-terminal prodomain of the NaD1 defensin of* Nicotiana alata* is sufficient for vacuolar targeting and plays an important role in detoxification of the defensin [65].

Plant defensins inhibit the growth of a wide range of fungi and in a lesser extent are toxic to mammalian cells or plants [66]. The proposed mechanism of action of plant defensins is to either destabilize the cell membrane by coating its outer surface or insert themselves into the membrane to form open pores allowing vital biomolecules to leak out of the cell [34, 64].

### 4.1. Cytotoxic and Anticancer Activity of Plant Defensins

In addition to the antifungal activities, plant defensins exhibit anticancer and cytotoxic effects (Table 3). The first plant defensin reported with anticancer activity was the defensin sesquin from* Vigna sesquipedalis* that inhibited the proliferation of MCF-7 and leukemia M1 (2.5 mg/mL) cells [44]. Furthermore, Wong and Ng [41] reported that the defensin limenin (0.1 mg/mL), a defensin from* Phaseolus limensis*, differentially inhibited the proliferation of leukemia cells, reaching 60% inhibition for M1 and 30% inhibition for L1210 cells; however, its effect against normal cells was not evaluated. Another plant defensin with effects on cancer cell is lunatusin, a defensin purified from the seeds of the Chinese lima bean (*Phaseolus lunatus* L.), which inhibited the proliferation of MCF-7 cells (IC_50_ 5.71 *μ*M). Unfortunately, lunatusin also possesses cell-free translation-inhibitory activity in the rabbit reticulocyte lysate system [45]. This indicates that this defensin may be cytotoxic to normal tissues and other cell types. However, from all the defensins studied, lunatusin is the only plant defensin with this effect.

Further studies identified other plant defensins that inhibit the proliferation of cancer cells, including breast and colon cancer, without cytotoxic effects on normal cells. A defensin from the purple pole bean (*Phaseolus vulgaris* cv. “Extra-long Purple Pole bean”) inhibited the proliferation of the cancer cell lines HepG2, MCF-7, HT-29, and Sila (IC_50_ 4–8 *μ*M) but did not affect human embryonic liver cells or human erythrocytes under the same conditions [46]. By contrast, coccinin from small scarlet runner beans (*Phaseolus coccineus* cv. “Major”), a peptide of 7 kDa and an N-terminal sequence resembling those of defensins, inhibited the proliferation of HL60 and L1210 cells (IC_50_ 30–40 *μ*M); however, it did not affect the proliferation of mouse splenocytes [47]. Similarly, phaseococcin from* P. coccineus* cv. “Minor” inhibited the proliferation of HL60 and L1210 cells (IC_50_ 30–40 *μ*M). This defensin did not affect the proliferation of mouse splenocytes or protein synthesis in a cell-free rabbit reticulocyte lysate system [48]. The lack of adverse effects of both of these defensins on the proliferation of isolated mouse splenocytes indicates that these molecules are selective. Finally, the conditioned media from bovine endothelial cells that express the cDNA of the defensin *γ*-thionin from* Capsicum chinense* inhibited 100% of the viability of HeLa cells but did not affect immortalized bovine endothelial cells [49]. Data from our laboratory indicate that this chemically synthetized defensin has a similar effect on both cells (data not published).

In general, the anticancer activity mechanism of plant defensins is poorly understood. However, Poon et al. [67] described the mechanism of the NaD1 defensin on the monocytic lymphoma cells U937. Interestingly, this effect was produced by a novel mechanism of cell lysis in which NaD1 acts via direct binding to the plasma membrane phospholipid phosphatidylinositol 4,5-bisphosphate (PIP_2_).

Thus, the anticancer activities of plant defensins suggest that these AMPs may be an alternative therapy for cancer treatment. The isolation and characterization of these peptides has increased, which allows for the identification of sequences that exhibit desirable characteristics against cancer cells.

## 5. Cyclotides

Cyclotides are macrocyclic peptides (~30 amino acids) with diverse biological activities, isolated from the Rubiaceae and Violaceae plant families. These molecules constitute a family of plant AMPs, members of which contain six conserved cysteines that stabilize the structure by the formation of disulfide bonds [74]. Cyclotides have a cystine knot with an embedded ring in the structure formed by two disulfide bonds and connecting backbone segments threaded by a third disulfide bond. These combined features of the cyclic cystine knot produce a unique protein fold that is topologically complex and has exceptional chemical and biological stability with pharmaceutical and medicinal significance for drug design [75].

**Figure 2:**
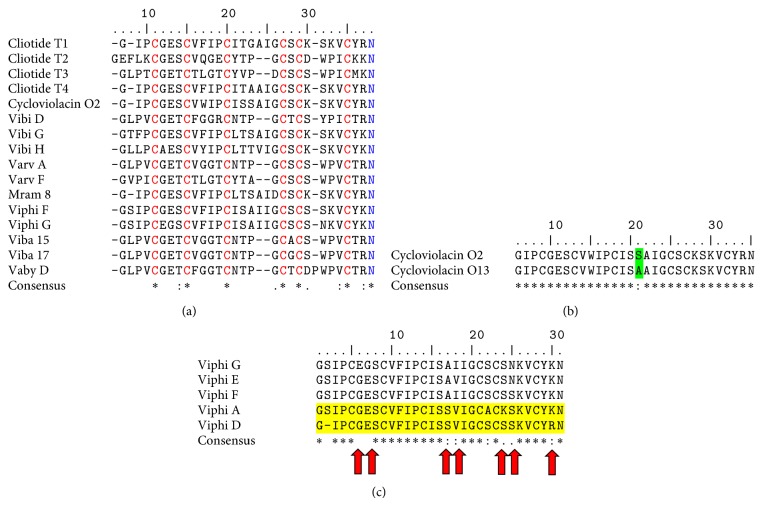
Alignment of amino acid sequences from cytotoxic cyclotides. (a) The cysteine residues present in all sequences and relevant to the classification are indicated in red letters. The asparagine residues present in all sequences and relevant to the cyclization process are indicated in blue letters. (b) Amino acid sequence alignment of cycloviolacins O2 and 13. The replacement of serine by alanine (shaded in green) increases the hemolytic effect by more than 3-fold. (c) Amino acid sequence alignment of Viphi G, Viphi E, Viphi F, Viphi A, and Viphi D cyclotides. Shaded in yellow are the sequences with no-toxic effects; the red arrows indicate the residues with specific variations. The sequences included in the alignment were cliotides T1 (GenBank accession AEK26402), T2 (GenBank accession AEK26403), T3 (GenBank accession AEK26404), and T4 (GenBank accession AEK26405) from* Clitoria ternatea*, cycloviolacins O2 (GenBank accession P58434) and O13 (GenBank accession Q5USNB) from* Viola odorata*, Vibi D (GenBank accession P85242), Vibi G (GenBank accession P85245), and Vibi H (GenBank accession P85246) from* Viola biflora*, Varv A (GenBank accession Q5USN7) and Varv F (GenBank accession 3E4H_A) from* Viola odorata*, Mram 8, Viphi A, Viphi D, Viphi E, Viphi F, and Viphi G [73] from* Viola philippica,* and Vaby D [55] from* Viola abyssinica*.

Cyclotides are biosynthesized ribosomally as a precursor protein that encodes one or more cyclotide domains. The arrangement of a typical cyclotide precursor protein is an endoplasmic reticulum signal sequence, a prodomain, a mature cyclotide domain, and a C-terminal region [76]. Although the excision and cyclization processes that yield cyclic mature peptides from these precursors are not fully understood, it has been suggested that asparaginyl endoproteinase enzyme activity plays an important role in this process [77]. This hypothesis is consistent with the presence of a conserved Asn (or Asp) residue at the C-terminus of the cyclotide domain within the precursor proteins (Figure 2(a)). It is also supported by studies of the expression of mutated cyclotides in transgenic plants, in which substitution of the conserved Asn by Ala abolished the production of cyclic peptides* in planta* [78].

The main role attributable to cyclotides is host defense, and there are molecules that are expressed in large quantities in the plant (up to 1 g/kg of leaf material) [75]. Furthermore, cyclotides display a wide range of biological and pharmacological activities, including anti-HIV, anthelmintic, insecticidal, antimicrobial, and cytotoxic effects [79]. Therefore, there is increasing interest in exploring the plant kingdom to identify new cyclotides.

### 5.1. Cytotoxic and Anticancer Activity of Cyclotides

One of the first activities reported for cyclotides was hemolytic activity, which only occurs in the cyclic condition. Cyclotides lose their hemolytic activity when they are linearized [80], demonstrating that the cyclic backbone is important for this activity, which also appears to be important for the other activities of cyclotides. A directed mutational analysis of cyclotide kalata B1, in which all 23 noncysteine residues were replaced with alanine, shows that both the insecticidal and hemolytic activities are dependent on a well-defined cluster of hydrophilic residues on one face of the cyclotide. Interestingly, these molecules retain the characteristic stability of the framework [73]. In addition, it has been suggested that the hemolytic activity of the cyclotides depends on the amino acid sequence. The cyclotides cycloviolacins O2 and O13 from* Viola odorata* have different hemolytic activities. Both molecules differ only in one residue (Figure 2(b)). Cycloviolacin O2 has a serine residue, whereas cycloviolacin O13 has an alanine in the same position. The loss of the hydroxyl group changes the hemolytic activity by more than 3-fold [50].

In general, cyclotides also show anticancer activity against human cancer cells (Table 4); however, two cyclotides from* Viola philippica* (Viphi D and Viphi E) did not show activity against the human gastric cancer BGC-823 cell line [51]. These peptides have similar sequences to the cyclotides Viphi F and Viphi G (Figure 2(c)), indicating that even minimal sequence changes can significantly influence the bioactivity. It has been suggested that the potency and selectivity of cyclotides is dependent on their primary structure. For example, a single glutamic acid plays a key role in the anticancer activity of cycloviolacin O2, and when this residue is methylated, a 48-fold decrease in potency is observed [52].

Cycloviolacin O2 from* Viola odorata* is particular promising because of its selective toxicity to cancer cell lines relative to normal cells, which indicates the possibility of its use as an anticancer agent [53]. Analysis of the proposed mechanism of action of this cyclotide shows that the disruption of cell membranes plays a crucial role in the cytotoxicity of cycloviolacin O2 because the damage to cancer cells (human lymphoma) can be morphologically distinguished within a few minutes, indicating necrosis [54]. However, this activity was not detected when this cyclotide was tested in a mouse tumor model. The reasons of this discrepancy are not fully understood, although high clearance rates or poor distribution to the site of action may be involved. Cycloviolacin O2 was also lethal to mice (2 mg/kg), but no signs of discomfort to the animals were observed at 1.5 mg/kg [55]. Recently the cyclotide MCoTI-I was engineering and the resulting cyclotide MCo-PMI showed activity* in vivo* in a murine xenograft model with prostate cancer cell; treatment (40 mg/kg) significantly suppressed tumor growth [81]. In the same way, HB7 cyclotide from* Hedyotis biflora* in an* in vivo* xenograft model significantly inhibited the tumor weight and size compared to control [82]. These results suggest that cyclotides may have a good anticancer bioactivity.

With respect to the action mechanism of cyclotides, a study showed that cycloviolacin O2 and kalatas B1–B9 target membranes through binding to phospholipids containing phosphatidylethanolamine headgroups [90]. Therefore, the biological potency of these cyclotides may be correlated with their ability to target and disrupt cell membranes. The knowledge of their membrane specificity could be useful to design novel drugs based on the cyclotide framework, allowing the targeting of specific peptide drugs to different cell types.

## 6. Small Cationic Peptides Isolated from Plants with Anticancer Activity

In addition to plant AMPs, other small linear and cyclic peptides (2–10 aa) with anticancer activity have been reported in plants. For example, the linear peptide* Cn*-AMP1, isolated and purified from coconut water (*Cocos nucifera*), was tested against Caco-2, RAW264.7, MCF-7, HCT-116 cells, and human erythrocytes and showed a reduction of cell viability in cancer cells without causing hemolysis [91]. Other examples are the peptides Cr-ACP, isolated from* Cycas revoluta*, and the acetylated-modified Cr-AcACP1, both repressors of cell proliferation of human epidermoid cancer (Hep2) and colon carcinoma. These peptides induce cell cycle arrest at the G0-G1 phase of Hep2 cells [92]. Moreover, four small cyclic peptides, dianthins C–F, have anticancer activity against Hep G2, Hep 3B, MCF-7, A-549, and MDA-MB-231 cancer cell lines (IC_50_ 20 *μ*g/mL) [93]. Furthermore, the cyclic heptapeptide cherimolacyclopeptide C, obtained from a methanol extract of the seeds of* Annona cherimola*, exhibited significant* in vitro* cytotoxicity against KB cells (IC_50_ 0.072 *μ*M) [94]. Other examples of small cyclic peptides are RA-XVII and RA-XVIII from the roots of* Rubia cordifolia* L., which have cytotoxicity against P-388 cells at 0.0030 *μ*g/mL and 0.012 *μ*g/mL, respectively; however, it was not determined whether these peptides are effective against normal cells [95]. Recently, an antiproliferative cyclic octapeptide (cyclosaplin) was purified from* Santalum album* L. The anticancer activity from this peptide was tested against human breast cancer (MDA-MB-231) cells and exhibited significant growth inhibition in a dose and time dependent manner (IC_50_ 2.06 *μ*g/mL). Additionally, cytotoxicity on normal fibroblast cell line at concentrations up to 1000 *μ*g/mL was not detected [56].

## 7. Conclusion and Future Perspectives

The identification and development of plant AMPs with anticancer properties will provide good opportunities for cancer treatment. AMPs with anticancer activities, including plant-derived peptides, show many therapeutic challenges that must be considered before they can be developed commercially. Strategies to solve their poor stability and susceptibility to proteolytic digestion, such as amino acid substitution, structural fusion of functional peptides, and conjugation with chemotherapeutic drugs, must be evaluated. Despite these limitations, AMPs are an important source of molecules useful for the design of new drugs. In this sense, cationic peptides from plants have great potential as anticancer agents, particularly because of their selectivity towards cancer cells, as has been demonstrated to coccinin and phaseococcin. The number of plant AMPs with anticancer activity is increasing and is expected to rise in the next years, particularly when the remaining plant AMP families are assessed. A crucial step in the studies of plant AMPs as anticancer agents is the identification of their mechanisms of action to discover new targets. Furthermore, the development of novel synthetic analogs of these natural molecules could enhance their activities, facilitating the development of new drugs. With the rapid development in proteomics, bioinformatics, peptide libraries, and modification strategies, these plant AMPs emerge as novel promising anticancer drugs in future clinical applications.

## Figures and Tables

**Table 1:** Classification of plant AMPs^1^.

Family	Disulfide bonds	Activity
Thionins	3-4	Bacteria, fungi, and cytotoxic
Defensins	3-4	Bacteria, fungi, and cytotoxic
Cyclotides	3	Bacteria, virus, insects, and cytotoxic
Knottin-like	3	Gram (+) bacteria and fungi
Shepherdins	0 (linear)	Bacteria and fungi
MBP-1	2	Bacteria and fungi
Ib-AMPs	2	Gram (+) bacteria and fungi
LTP	3-4	Bacteria and fungi
Snakins	6	Bacteria and fungi
Hevein-like	4	Gram (+) bacteria and fungi
*β*-Barrelins	6	Fungi
2S albumins	2	Bacteria and fungi

^1^Modified from [21–23].

**Table 2:** Thionins with anticancer and cytotoxic activity.

Name	Species	Activity against	Cytotoxic activity	Anticancer activity	Reference
Pyrularia	*Pyrularia pubera *	B16, HeLa, rat hepatocytes, and lymphocytes	Yes	Yes	[24]

Viscotoxin B2	*Viscum coloratum *	Rat sarcoma cells	Not tested	Yes	[25]

Viscotoxins 1-PS, A1, A2, A3, and B	*Viscum album *	Human lymphocytes	Yes	Not tested	[26]

Viscotoxin C1	*Coloratum ohwi *	Rat sarcoma cells	Not tested	Yes	[27]

Ligatoxin B	*Phoradendron liga *	U-937-GTB ACHN	Not tested	Yes	[28]

Ligatoxin A	*Phoradendron liga *	Animal cells	Yes	Not tested	[29]

Phoratoxins A and B	*Phoradendron tomentosum *	Mice	Yes	Not tested	[30]

Phoratoxins C, D, E, and F	*Phoradendron tomentosum *	10 cancer cell lines	Not tested	Yes	[31]

Thi2.1	*Arabidopsis thaliana *	HeLa, A549, MCF-7, and bovine mammary epithelial cells	Yes	Yes	[32]

*β*-Purothionin	*Tricum aestivum *	p388	Not tested	Yes	[33]

**Table 3:** Plant defensins with anticancer and cytotoxic activity.

Name	Species	Activity against	Cytotoxic activity	Anticancer activity	Reference
Sesquin	*Vigna sesquipedalis *	MCF-7 and M1	Not tested	Yes	[44]

Limenin	*Phaseolus limensis *	L1210 and M1	Not tested	Yes	[41]

Lunatusin	*Phaseolus lunatus *	MCF-7 rabbit reticulocyte	Yes	Yes	[45]

Purple pole defensin	*Phaseolus vulgaris* cv. “Extra-long Purple Pole bean”	HepG2, MCF7, HT-29, and SiHa	No	Yes	[46]

Coccinin	*Phaseolus coccineus* cv. “Major”	HL60 and L1210	No	Yes	[47]

Phaseococcin	*Phaseolus coccineus *	L1210 and HL60	No	Yes	[48]

*γ*-Thionin	*Capsicum chinense *	HeLa	No	Yes	[49]

NaD1	*Nicotiana alata *	U937	Not tested	Yes	[67]

Mitogenic defensin	*Phaseolus vulgaris *	MCF-7, murine splenocytes	Yes	Yes	[68]

Vulgarinin	*Phaseolus vulgaris *	MCF-7, L1210, and M1	Not tested	Yes	[69]

Cloud bean defensin	*Phaseolus vulgaris* cv. cloud bean	L1210 and MBL2	Not tested	Yes	[70]

Nepalese	*Phaseolus angularis *	L1210, MBL2	Not tested	Yes	[71]

Gymnin	*Gymnocladus chinensis* Baill	M1, HepG2, and L1210	Not tested	Yes	[72]

**Table 4:** Cyclotides with anticancer and cytotoxic activity.

Name	Species	Activity against	Cytotoxic activity	Anticancer activity	Reference
Cycloviolacin O2	*Viola odorata *	U-937, HeLa	Yes	Yes	[54]

Viphi A, Viphi F, and Viphi G	*Viola philippica *	MM96L, HeLa, BGC-823, and HFF-1	Yes	Yes	[51]

MCoTI-I	*Momordica cochinchinensis *	LNCaP and HCT116	Not tested	Yes	[81]

HB7	*Hedyotis biflora *	Capan2 and PANC1	Not tested	Yes	[82]

Vaby A and Vaby D	*Viola abyssinica *	U-937	Not tested	Yes	[83]

Cliotides T1–T4	*Clitoria ternatea *	HeLa and human erythrocytes	Yes	Yes	[84]

Psyle A, Psyle C, and Psyle E	*Psychotria leptothyrsa *	U-937	Not tested	Yes	[85]

Vibi G and Vibi H	*Viola biflora *	U-937	Not tested	Yes	[86]

Varv A and Varv F	*Viola arvensis *	10 cancer cell lines	Not tested	Yes	[87]

Viba 15, Viba 17, and Mram 8	*Viola philippica *	HFF1, MM96L, HeLa, BGC-823, and HFF-1	Yes	Yes	[51]

CT-2, CT-4, CT-7, CT-10, CT-12, and CT-19	*Clitoria ternatea *	A549	Not tested	Yes	[88]

Kalata B1 and kalata B2	*Oldenlandia affinis *	U-937 GTB HT-29 Ht116	Yes	Yes	[89]
